# Retraction: Hypothalamic Inhibition of Acetyl-CoA Carboxylase Stimulates Hepatic Counter-Regulatory Response Independent of AMPK Activation in Rats

**DOI:** 10.1371/journal.pone.0295063

**Published:** 2023-11-27

**Authors:** The PLOS ONE Editors

After this article [1] was published, concerns were raised about some of the western blots in Figs. 1 and 5. Specifically:

- In Fig. 1C, there appear to be vertical discontinuities between lanes 4 and 5 in the top anti-GAPDH panel and between lanes 3 and 4 in the bottom anti-ACC panel.
- Lanes 1–4 in the IB:anti-ACC panel in Fig. 1B, lanes 3–6 in the IB:anti-PEPCK panel in Fig. 5A, and lanes 3–6 in the IB:anti-p-ACC panel in Fig. 5B appear similar.
- The IB:anti-GAPDH panel in Fig. 5A in [1] appears similar to the IB:AKT panel in Fig 3C in [2].

During editorial follow up on these issues, the corresponding author stated that the original data underlying this article [1] are no longer available and they are unable to properly assess the experimental results presented in each figure.

In light of the above issues, which could not be resolved in the absence of the original underlying data, the *PLOS ONE* Editors retract this article.

MAT agreed with the retraction. GAS, VDP, LIS, DCV, RFdM, DSR, AST and LAV either did not respond directly or could not be reached. EAFRR did not respond to the editorial decision.

Owing to the concerns about similarities with previously published content [2], published 2011 by Society for Experimental Biology and Medicine [2], which is not offered under a CC-BY license, the IB:anti-GAPDH panel in Fig. 5A is excluded from this article’s [1] license. At the time of retraction, the article [1] was republished to note this exclusion in the Fig. 5 legend and the article’s copyright statement.
